# Risk salience of a novel virus: US population risk perception, knowledge, and receptivity to public health interventions regarding the Zika virus prior to local transmission

**DOI:** 10.1371/journal.pone.0188666

**Published:** 2017-12-21

**Authors:** Rachael Piltch-Loeb, David M. Abramson, Alexis A. Merdjanoff

**Affiliations:** College of Global Public Health, New York University, New York, NY, United States of America; Johns Hopkins University, UNITED STATES

## Abstract

**Background:**

As the incidence of Zika infection accelerated in Central and South American countries from November 2015 through April 2016, U.S. public health officials developed vector control and risk communication strategies to address mosquito-borne and sexual modes of transmission. This study reports upon U.S. perceptions of the Zika virus prior to domestic transmission, and analyzes the association of socio-economic, political, knowledge and risk factors with population receptivity to selected behavioral, environmental, and clinical intervention strategies.

**Methods:**

A representative sample of 1,233 U.S. residents was drawn from address-based telephone and mobile phone lists, including an oversample of 208 women of child-bearing age living in five U.S. southern states. Data were collected between April and June, 2016, and weighted to represent U.S. population distributions.

**Results:**

Overall, 78% of the U.S. population was aware of Zika prior to domestic transmission. Those unaware of the novel virus were more likely to be younger, lower income, and of Hispanic ethnicity. Among those aware of Zika, over half would delay pregnancy for a year or more in response to public health warnings; approximately one third agreed with a possible vector-control strategy of targeted indoor spraying by the government; and nearly two-thirds agreed that the government should make pregnancy-termination services available to women who learn their fetus had a Zika-related birth defect. Receptivity to these public health interventions varied by age, risk perception, and knowledge of the virus.

**Conclusion:**

Risk salience and population receptivity to public health interventions targeting a novel virus can be conditioned on pre-existing characteristics in the event of an emerging infectious disease. Risk communicators should consider targeted strategies to encourage adoption of behavioral, environmental, and clinical interventions.

## Introduction

The emergence of a novel virus strain such as Zika virus (ZKV) offers an opportunity to examine how the public perception of risk salience and receptivity to public health interventions evolve as a health threat approaches. Since 2001, the United States has experienced or anticipated a number of novel biological threats, including weaponized anthrax, SARS, H5N1, H1N1, MERS, and Ebola, among others. These cases have generally begun with maximal uncertainty about the agent’s infectiousness, virulence, transmission pathways, vectors, and health outcomes [1]. Scientific certainty and epidemiological evidence may accumulate over the course of weeks, as in the case of anthrax [2], or months, as in the cases of SARS and H1N1 [1, 3–5], but the development or identification of effective medical countermeasures often lag significantly behind the curve of the epidemic. In such cases, public health relies upon enhanced surveillance strategies, non-pharmaceutical interventions, and broad scale prevention campaigns. The success of these activities often depends upon public receptivity and political support [4, 6].

Enlisting the public’s support for public health interventions is generally accomplished by focusing on the potential health risks associated with the emerging infectious agent and promoting the efficacy of the proposed interventions. This parallel attention to risk and efficacy is the foundation of a number of behavior change and risk communication models, including the Health Belief Model [7], the Extended Parallel Process Model [8], and the Protective Action Decision Model [9]. Each of these models assumes that the salience of a risk increases as the target population appreciates its susceptibility to the agent and the severity of the consequences. Furthermore, the greater the dread and the immediacy of the threat, the likelier the public is to take preventive actions [10]. Acceptance of public health activities is built upon the salience of the risk to the public and a variety of factors at the individual and societal level However, in the event of an emerging biological threat, public health officials are often confronted with the challenge of informing the public, conducting surveillance, and, and developing prevention campaigns in the face of considerable uncertainty. During an emerging infectious disease response, public health authorities rely on the willingness of the general public to adhere to preventive public health measures to limit the consequences of the event, yet the compliance with recommended precautionary behaviors has been inconsistent at best. Since much of the public’s willingness may be dependent upon its appreciation of its risk and the efficacy of public health interventions, it is incumbent upon public health officials and risk communicators to appreciate the factors that may influence these perceptions.

### The Zika experience

Despite the number of outbreaks occurring within the last two decades, limited research has been conducted on the factors that affect people’s decision-making and behavior when confronted with an emerging infectious disease outbreak in real time [11–13]. This is in part attributable to the challenges of doing prospective research as a disease emerges [14]. The Zika epidemic provides the latest example to explore the potential predictors of health behavior change in an emerging infectious event. Over the span of six months between October 2015 and March 2016 the Zika virus spread throughout parts of South America, Central America, and the United States. As of April 2016 there were 5,222 cases of Zika reported in the U.S., and over 36,000 additional cases in U.S. territories [15]. Parallel to the epidemiology, there was a rapid increase in scientific knowledge about the viral etiology and biological mechanism, as well as increasing clarity about the clinical association of Zika and various newborn health effects. The health consequences associated with Zika among newborns include microcephaly, neurological deficits such as cognitive disabilities and blindness, and infant and fetal death [16, 17]. Given the absence of either a vaccination or a medical treatment for the viral infection, U.S. public health efforts have focused on non-pharmaceutical interventions that include behavioral strategies (i.e., insecticide use; not traveling to Zika-endemic regions; condom use; and delaying pregnancy), environmental strategies (i.e., vector control efforts based upon window screening; wide-area spraying; and larvacide deployment), and clinical strategies (i.e., availability of and access to pregnancy termination services; blood supply screening; and targeted patient testing) [18]. Communicating the risks of Zika and conveying the merits and options associated with these various interventions has become a central focus of public health, especially as officials considered the potential for large-scale exposure of the U.S. population, and ramifications for pregnant women.

### Theoretical framework

**Fig 1:**
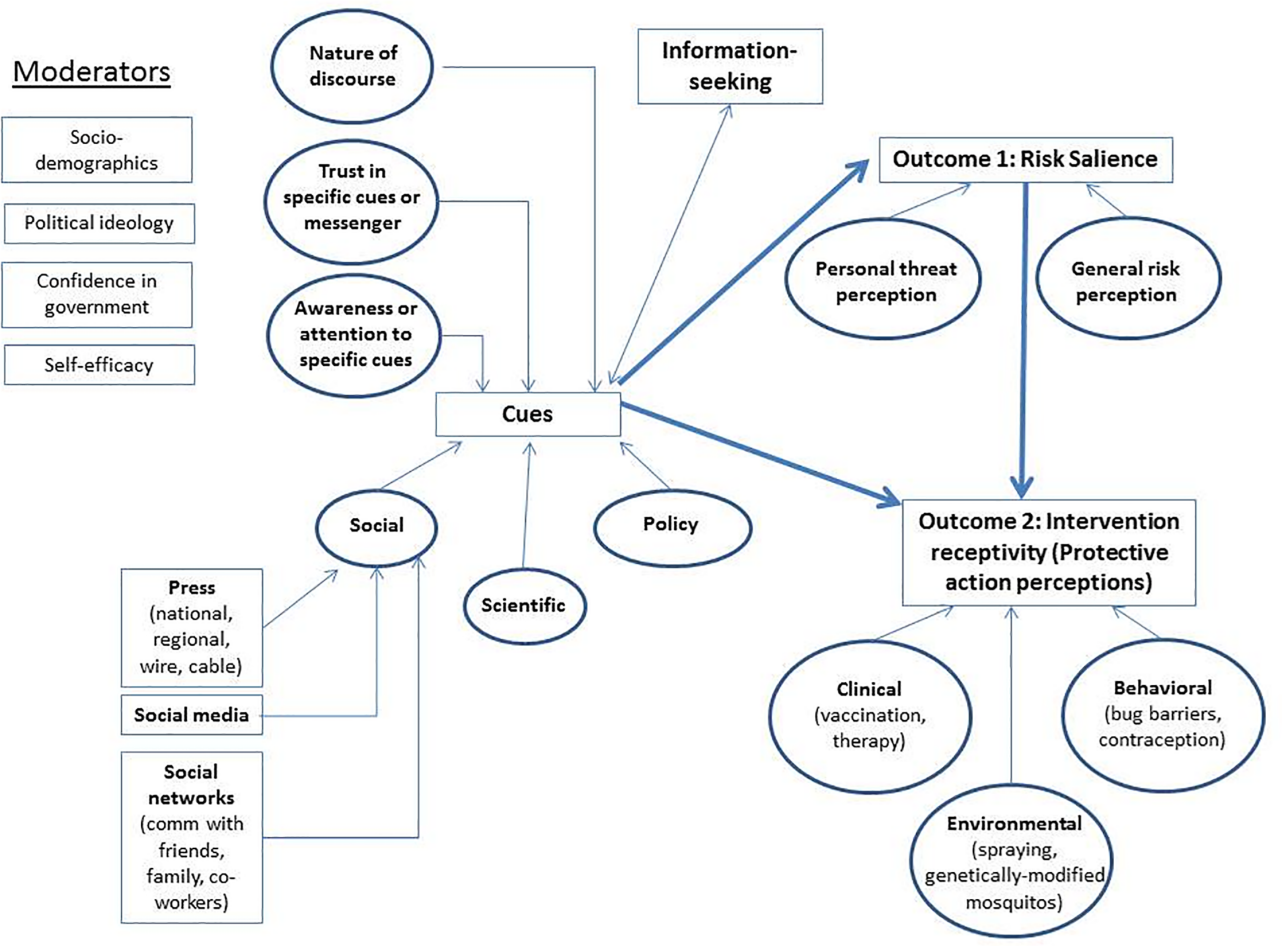
Evolving risk salience framework.

As noted above, prior theories of risk communication have focused upon several essential elements associated with behavior change: risk salience, which includes an appraisal of the proximity, severity, and dread associated with the hazard, self-efficacy, and knowledge of the threat and its consequences [8, 19–21]. Further, theorists have highlighted the relevance of such external factors as social networks, media, and other informational cues that can influence risk salience and behavior change [9, 22, 23]. To examine how risk perception, knowledge, and informational cues related to intervention, a theoretical model was developed that adapts the Protective Action Decision Model developed by Lindell and Perry and incorporates cues that impact knowledge and risk, such as source of information and policy environment (Fig 1) [9].

Drawing on this model, this analysis reports the patterns of U.S. population knowledge and risk perception of the Zika virus; population receptivity to various clinical, behavioral, and environmental interventions; and the relationship among knowledge, risk perception, and intervention receptivity.

## Methods

A structured telephone survey of 1,233 US residents was conducted between April 15 and May 18, 2016, beginning approximately three months after WHO declared Zika a public health emergency. Data was collected by SSRS Omnibus Survey, a national, weekly, dual-frame bilingual telephone survey. Each weekly wave of the Omnibus survey includes 1,000 interviews of which 600 are obtained on cell phones, and approximately 35 are complete in Spanish. Questions designed by the research question took approximately eight minutes to ask. Sample telephone numbers are computer generated and loaded into an on-line sample file accessed directly by a computer-assisted telephone interviewing (CATI) system. The landline sample is structured through MSG Genesys database using eighteen independent strate, comprised of the nine census divisions, split by metro and non-metro county definitions. Calls were conducted weekly between Wednesday and Sunday, The AAPOR RR3 for the general sample was 4%, while the AAPOR RR3 for the oversample was 6%, for an average AAPOR R3 of 4.4%. Sampling protocol called for a maximum of 15 call attempts on all sample. The maximum number of calls on completes was nine. The average number of call attempts for completes was seven. A total of 277,427 calls were made to reach 1025 completes in the general sample.

The data were weighted to represent the U.S. adult population. Weighting procedures were the following. All respondents were asked detailed geographic questions including census region, state, metropolitan statistical area, designated market area, and metro status based on being asked to provide their Zipcode. Post stratification iterative proportional fitting was used to balance the sample to known adult population parameters based on the most recent March Supplement of the US Census Bureau’s Current Population Survey. The sample was post-stratified and balanced by age, race/ethnicity, sex, region, education, marital status, population density, and phone-usage. Weighting procedures took into account the disproportionate probabilities of household and respondent selection due to the number of separate telephone landlines and cellphones answered by respondents and their households.

The sample frame included an oversampling of women of child-bearing age between the ages of 18–45 living in the southern tier states of Florida, Alabama, Mississippi, Louisiana, and Texas. Nine waves of the Omnibus weekly survey were used to collect the oversample of Women of child bearing age. The same distribution of landline and cellphones were used to make the calls. Oversample respondents were added to the general sample and weighted in the same way.

At the time of the survey, 157 pregnant women residing in U.S. states and the District of Columbia had been reported as likely Zika infections with another 122 in U.S. Territories [24].

### Measures

#### Outcome measures

Public receptivity to three potential public health campaigns described in Fig 1 were assessed: recommendations to delay pregnancy for a year; government-run indoor spraying of insecticides; and federal support for pregnancy termination services for Zika-infected pregnant women at risk of carrying a fetus with birth defects. Respondents were asked whether or not they supported each intervention based on a four-point Likert scale. For example in regard to delay in pregnancy, respondents were asked, “In terms of actions you might take yourself, how likely would you or your partner be to delay pregnancy for a year or more?” Response options included very likely, somewhat likely, somewhat unlikely, and very unlikely). Though females bear the direct risks of pregnancy their partners are often influential in their healthcare and health decisions before, during, and after pregnancy. Therefore, questions regarding pregnancy delay were asked of all genders. Receptivity was then dichotomized so that those who agreed or highly agreed with the intervention were coded as receptive and those who did not agree or highly disagreed were coded as not receptive to the intervention, which served as the reference category. These specific interventions were selected as examples of a preventative behavioral strategy (e.g., pregnancy), environmental strategy (e.g., spraying), and clinical strategy (e.g., pregnancy termination).

#### Independent variables

Awareness of Zika was based on response to the question, “Are you aware of the Zika virus?” and was dichotomized as aware (yes = 1) or not aware (no = 0). For those who reported that they were aware, respondents were coded as knowledgeable if they knew that the virus could be sexually transmitted, could be carried asymptomatically, and could cause birth defects. Perceptions of personal risk and community risk were based on self-report of respondents, and were dichotomized (1 = at risk; 0 = not at risk). Respondents identified sources of information they accessed for information on Zika from a closed survey question and also reported which was their primary source. For analytic purposes, the eight primary sources were recoded into four categories: 1) informal sources, which encompassed social media, family, and friends; 2) formal news sources, which included print, online, and broadcast news; 3) personal physicians; and, 4) government communications.

#### Control variables

All control variables were based on responses to survey items included in the SSRS weekly Omnibus. Control variables include demographic measures, such as age (18–29 (ref.); 30–45; 46–64; 65+), gender (male (ref.); female), race (Non-Hispanic White (ref.); Non-Hispanic Black; Hispanic; Other), household income (less than $25,000 (ref.); $25,000–49,999; $50,000–99,999; more than $100,000), education (less than high school (ref.); HS/GED; some college; 4-year college) political views (Republican (ref.); Democrat; Independent), and region (Gulf Coast (ref.); Mid-US; North),. Region was split into the following categories, which we have referred to as Gulf Coast (TX, AL, MS, LA, FL), Mid-US (AZ, AR, CA, CO, DE, GA, HI, IL, IN, KS, KY, MD, MO, NE, NV, NM, NC, OK, SC, TN, UT, VA), and North-US (AK, CT, ID, IA, ME, MA, MI, MN, NH, NJ, NY, ND, OH, OR, PA, RI, SD, VT, WA, WI, WY). Political views were added in particular due to the often political nature of pregnancy- termination related decisions.

#### Analytic plan

We began analyses by exploring bivariate demographic differences based on awareness of the Zika virus (N = 1,233). Following this initial exploration, in order to examine any associations between knowledge, risk, and intervention receptivity analyses only considered those aware of the Zika virus (N = 1,004). Researchers examined bivariate associations for differences among those receptive and not receptive to each intervention. The team then conducted unadjusted and adjusted logistic regression to test the association of knowledge and risk perception with each public health intervention. The model included controls for demographic, socio-economic, regional, and political views, as bivariate analyses suggested that these factors likely relate to intervention receptivity and potentially influence the relationship between knowledge or risk, and receptivity. Our analysis operationalized all elements of our theoretical model including all moderators we measured, informational cues, risk perception, knowledge, and intervention receptivity. Based on our theoretical framing and our review of the literature, we kept all model elements in our multivariable models, as the effect or lack of effect of each predictor, has implications for public health practice. In this paper we do not attempt to theoretically operationalize our model, and have treated potential moderators and confounders the same way in our analyses to understand the relationship between risk, knowledge, and intervention receptivity.

All analyses used weighted data with significance levels set at the p<0.01 level. Respondents who did not provide an answer to a survey question were coded as refused or don’t know. Those who refused or replied don’t know were changed to missing for analytic purposes. Bivariate associations are among those who responded to the original survey questions. Multivariable models were run on complete cases only. Researchers conducted analyses using Stata statistical software, version 14. The Institutional Review Board of New York University approved the research.

### Results

Table 1 presents the weighted bivariate associations between Zika awareness and covariates. Overall, nearly 78% of all respondents indicated that they were aware of Zika. There is a statistically significant difference for Zika awareness amongst age groups. Of those aware of Zika, more than one third (34.4%) were 46–64 years old. Other age groups’ awareness of ZKV were lower, as only 17.9% of those aware of Zika were 18–29 years old, 27.1% were 30–45 years old and 20.6% were 65 and older. The youngest age group (18–29) reported the highest rates of unawareness with 34.4%. Unawareness of Zika decreased with age, as 29.3 of those unaware of Zika were 30–45 years old, 22.4 were 46–64 years old and 13.9% were 65 and older. There were also statistically significant racial differences for Zika awareness. Of those aware of Zika, 68% were Non-Hispanic White, 10.3% were Non-Hispanic Black, 13.5% were Hispanic and 8.2% reported their race as Other. Of those unaware of Zika, 54.1% were Non-Hispanic White, 16.4% were Non-Hispanic Black, 21.6% were Hispanic, and 7.9 reported their race as Other. Differences in awareness were also statistically significant by household income level. Of those who were aware of Zika, 21.0% reported a household income above $100,000 a year, 29.5% reported income between $50,000–99,000, 25.3% reported income between $25,000–49,999, and 24.1% reported income below $25,000. Unawareness of Zika decreased by level of household income, with 47.7% of those who were unaware making $25,000 or less compared to 2.3% making over $100,000. There were also statistically significant differences by political party affiliation. Among those aware of Zika, 24.3% identified as Republicans, 37.2% identified as Democrats, and 38.5% identified as Independents. Among those unaware of Zika, 10.6% identified as Republicans, 40.8% identified as Democrats, and 48.6% identified as Independents. Lastly, there were statistically significant differences regarding awareness of Zika and level of education attained with 10.9% aware of Zika attaining less than a high school degree, 28.5% attaining a high school diploma or GED, 25.3% attaining some college or an associate’s degree, and 35.3% attaining a four year college degree or more. Of those who reported being unaware of Zika, 16.3% attained less than a high school degree, 51% attained a high school diploma or GED, 20.5% attained some college or an associate’s degree, and 12.2% attained a four year college degree or more.

**Table 1:** Weighted bivariate associations between Zika awareness and covariates.

	Aware of Zika	Not aware of Zika	Total
	77.90%	22.10%	100%
**Gender**	col%	col%	
Male	46.5	55.8	48.6
Female	53.5	44.2	51.4
Age***			
18–29	17.9	34.4	21.7
30–45	27.1	29.3	27.6
46–64	34.4	22.4	31.7
65+	20.6	13.9	19.1
**Region**			
Gulf Coast	20.1	19.4	20.0
Mid US	42.8	49.5	44.3
North	37.1	31.1	35.7
Race**			
Non-Hispanic White	68.0	54.1	64.9
Non-Hispanic Black	10.3	16.4	11.7
Hispanic	13.5	21.6	15.3
Other	8.2	7.9	8.1
Household Income***			
Less than $25,000	24.1	47.7	29.3
$25,000–49,999	25.3	31.2	26.6
$50,000–99,999	29.5	18.8	27.2
More than $100,000	21.0	2.3	16.9
Political Views**			
Republican	24.3	10.6	21.5
Democrat	37.2	40.8	38
Independent	38.5	48.6	40.6
Education Attained***			
Less than high school	10.9	16.3	12.1
High School Diploma/GED	28.5	51	33.5
Some college/Associates Degree	25.3	20.5	24.3
Four year college degree or more	35.3	12.2	30.2

** p < 0.01

*** p < 0.001 (+/-3% age points at the 95% confidence interval)

Table 2 presents weighted crude and multivariate logistic regressions for the association between Zika interventions and knowledge, risk perceptions and covariates. All covariates in our theoretical model were included in our multivariable analysis. Following each regression analysis, we also checked for multicollinearity to ensure covariates were not redundant.

**Table 2:** Weighted multivariate logistic regressions for the association between Zika interventions and knowledge, risk perceptions, and covariates among those aware of Zika.

	Crude Odds Ratio for Delay Pregnancy OR (CI)	Adjusted Odds Ration for Delay Pregnancy OR (CI)	Crude Odds Ratio for Indoor Spraying OR (CI)	Adjusted Odds Ration for Indoor Spraying OR (CI)	Crude Odds Ratio for Abortion Availability OR (CI)	Adjusted Odds Ratio for Abortion Availability OR (CI)
**Gender**						
Male	*ref*.	*ref*.	*ref*.	*ref*.	*ref*.	*ref*.
Female	1.14 (0.83, 1.57)	0.70 (0.42, 1.18)	0.69(0.51,0.94)*	0.86 (0.53, 1.38)	1.24(0.88,1.75)	1.32 (0.79, 2.22)
**Age**						
18–29	*ref*.	*ref*.	*ref*.	*ref*.	*ref*.	*ref*.
30–45	0.47 (0.29, 0.76)**	0.64 (0.29, 1.43)	0.90 (0.57,1.43)	0.69 (0.33, 1.42)	0.55 (0.31,0.98)*	0.25 (0.09, 0.63)**
46–64	0.56 (0.35, 0.87)*	1.10 (0.52, 2.32)	0.57 (0.37,0.85)*	0.49 (0.25, 0.96)*	0.70(0.40,1.21)	0.55 (0.23, 1.32)
65+	0.57 (0.34, 0.94)*	1.06 (0.48, 2.37)	0.51 (0.33,0.81)*	0.37 (0.17, 0.77)**	0.63(0.36,1.11)	0.67 (0.26, 1.73)
**Region**						
Gulf Coast	*ref*.	*ref*.	*ref*.	*ref*.	*ref*.	*ref*.
Mid US	0.81 (0.53, 1.23)	0.78 (0.40, 1.51)	1.03(0.70,1.50)	0.98 (0.54, 1.78)	1.28(0.82,2.01)	1.76 (0.93, 3.32)
North	0.75 (0.49, 1.16)	0.52 (0.26, 1.07)	1.08(0.73,1.60)	1.19 (0.66, 2.16)	1.60(1.01,2.53)	2.13 (1.08, 4.21)*
**Race**						
Non-Hispanic White	*ref*.	*ref*.	*ref*.	*ref*.	*ref*.	*ref*.
Non-Hispanic Black	1.32 (0.80, 2.19)	1.25 (0.56, 2.77)	1.25(0.77,2.03)	0.93 (0.44, 1.95)	1.41(0.77,2.60)	1.08 (0.45, 2.58)
Hispanic	2.17 (1.33, 3.54)	2.13 (0.84, 5.43)	1.76(1.15,2.71)*	1.29 (0.60, 2.78)	1.91(1.10,3.40)*	1.68 (0.66, 4.23)
Other	1.26 (0.69, 2.29)	2.14 (0.72, 6.37)	1.16(0.65,2.08)	0.45 (0.17, 1.18)	1.30(0.69,2.46)	1.09 (0.35, 3.40)
**Household Income**						
Less than $25,000	*ref*.	*ref*.	*ref*.	*ref*.	*ref*.	*ref*.
$25,000–49,999	1.01 (0.63, 1.63)	1.04 (0.46, 2.36)	0.87(0.55,1.36)	0.74 (0.36, 1.52)	0.58(0.33,1.02)	0.78 (0.34, 1.77)
$50,000–99,999	0.70 (0.44, 1.13)	0.65 (0.30, 1.38)	1.02(0.66,1.60)	0.82 (0.4, 1.60)	0.61 (0.36,1.05)	0.56 (0.24, 1.30)
More than $100,000	0.46 (0.27, 0.79)	0.48 (0.19, 1.23)	1.19(0.74,1.91)	0.88 (0.39, 1.99)	0.89(0.51,1.55)	1.89 (0.73, 4.87)
**Political Views**						
Republican	*ref*.	*ref*.	*ref*.	*ref*.	*ref*.	*ref*.
Democrat	1.52(0.97,2.36)	1.79 (0.88, 3.65)	1.62(1.08,2.41)*	1.35 (0.70, 2.59)	6.62(4.07,10.80)***	11.78 (5.45, 25.42)***
Independent	1.45(0.94,2.25)	1.40 (0.69, 2.84)	1.71(1.15,2.55)**	2.19 (1.15, .17)*	2.48(1.60, 3.85)***	3.28 (1.71, 6.32)***
**Education Attained**						
Less than high school	*ref*.	*ref*.	*ref*.	*ref*.	*ref*.	*ref*.
HS/GED	0.92 (0.52,1.61)	0.75 (0.25, 2.27)	0.50(0.30,0.85)*	0.53 (0.21, 1.35)	0.62(0.32,1.19)	0.56 (0.21, 1.50)
Some college	0.69 (0.39,1.20)	0.97 (0.33, 2.84)	0.60(0.36,1.0)*	0.60 (0.25, 1.44)	0.72(0.38,1.36)	1.03 (0.38, 2.79)
4 yr. college+	0.62 (0.36,1.07)	0.67 (0.22, 2.02)	0.94(0.58,1.54)	0.97 (0.41, 2.31)	0.68(0.37,1.24)	0.95 (0.35, 2.57)
**Confident government can address ZKV issue**						
No	*ref*.	*ref*.	*ref*.	*ref*.	*ref*.	*ref*.
Yes	1.36(0.97,1.92)	1.12 (0.62, 2.05)	1.87(1.35,2.59)***	1.52 (0.87, 2.66)	1.61(1.13,2.31)**	1.25 (0.74, 2.11)
**Knowledge people can have ZKV without symptoms**						
No	*ref*.	*ref*.	*ref*.	*ref*.	*ref*.	*ref*.
Yes	1.28(0.79,2.07)	1.31(0.66,2.61)	1.78(1.11,2.86)*	1.66 (0.87, 3.16)	1.30(0.81,2.08)	1.37 (0.70, 2.66)
**Knowledge ZKVcan be sexually transmitted**						
No	*ref*.	*ref*.	*ref*.	*ref*.	*ref*.	*ref*.
Yes	1.24(0.83,1.87)	1.15 (0.66, 2.00)	1.27(0.87,1.86)	1.31 (0.81, 2.13)	1.43(0.97,2.11)	1.43 (0.82, 2.49)
**Believe personally at risk for Zika**						
No	*ref*.	*ref*.	*ref*.	*ref*.	*ref*.	*ref*.
Yes	1.66(1.12,2.46)*	2.51 (1.31, 4.81)**	1.10(0.77,1.57)	1.13 (0.65, 1.96)	1.19(0.82,1.72)	1.49 (0.81, 2.73)
**Believe community at risk for Zika**						
No	*ref*.	*ref*.	*ref*.	*ref*.	*ref*.	*ref*.
Yes	1.42(0.98,2.06)*	0.94 (0.52, 1.71)	0.78(0.55,1.10)	0.80 (0.48, 1.33)	1.12(0.79,1.60)	1.20 (0.67, 2.15)
**Primary source of Information on Zika**						
Family/Friends/Social Media	*ref*.	*ref*.	*ref*.	*ref*.	*ref*.	*ref*.
News/TV/Radio	0.92 (0.59,1.44)	0.97 (0.45, 2.09)	0.61(0.40,0.93)*	0.65 (0.33, 1.31)	0.78(0.43,1.40)	1.48 (0.62, 3.51)
Doctor	0.89 (0.39,2.01)	25.80 (2.03, 327.09)*	0.60(0.26,1.34)	0.31 (0.06, 1.65)	0.53(0.12,2.40)	0.55 (0.06, 4.69)
Government	1.50(0.48,4.67)	2.83 (0.52, 15.45)	0.67(0.26,1.71)	0.99 (0.21, 4.69)	1.08(0.28,4.24)	1.80 (0.30, 10.83)

* p < 0.05

** p < 0.01

*** p < 0.001

Those who believe they are personally at risk for Zika or who spoke to a doctor about Zika were more likely to endorse pregnancy delay. Older individuals and those who identify as a Republican were least likely to endorse indoor, governmental-run fumigation. Regarding availability of pregnancy-termination services, only individuals identifying as Democratic or Independent in their political views, living in the North East region of the US, or who were between the ages of 3–45, were more likely to support this intervention.

## Discussion

Even at a reasonably early stage of this evolving biologic threat, there was a substantial proportion of the U.S. population who were aware of Zika’s association with microcephaly and who knew that the virus could be sexually transmitted [25]. However, there were significant gaps in awareness among non-White U.S. adults and those with lower levels of education, suggesting a need for public health campaigns to increase awareness targeted towards specific populations.

Of the behavioral, environmental, and clinical interventions considered, overall the population is most receptive to federal funding for pregnancy termination services (64% agree), moderately receptive to delay pregnancy for a year or more (55% agree), and least receptive to the government use of indoor spraying inside homes (34% agree).

Past predictors of health behavior change in an emerging infectious disease have included demographics such as gender, education, and ethnicity, risk perception, subjective norms, efficacy, and trust in authorities or message [12, 26–28]. These results are consistent with some of these findings, yet more nuanced. Depending on the intervention, distinct elements of the theoretical model were most relevant.

The large gap in receptivity to these three interventions, in conjunction with the variation in factors that are significantly associated with receptivity—ranging from demographics to political affiliation to risk perception—reflect a challenge for the U.S. public health community. Public health risk communication plays a central role in promoting and protecting the health and well-being of the public before, during, and after an infectious disease outbreak. Recommendations that are timely, clear, and actionable are essential to the prevention and mitigation of negative consequences. Public health educators and information officers must take care to promote effective messages to the public.

A one-size-fits-all approach to risk messaging designed to encourage intervention uptake in the event of a large-scale Zika outbreak is likely to have limited success. While there currently does not seem to be an effect of knowledge or risk on receptivity to indoor spraying or making pregnancy-termination services available for women affected by Zika, there is a strong and singular relationship between risk and receptivity to delaying pregnancy. If pregnancy delay continues to be one of the primary modes of mitigating Zika, messaging must highlight how individuals are at personal risk for Zika to encourage adherence to this recommendation. The result for pregnancy delay is consistent with emerging disease events, where risk perception was consistently found to be related to prospective behavior change following a cross-sectional survey during swine flu and predicted behavioral intention for preventive measures during H1N1 [29, 30].

This study provides a snapshot of attitudes towards Zika during a period of limited domestic cases, prior to mosquito season in the United States. Limitations of this study are the specific time frame in which the study was conducted, ability to only use dichotomous measures to explore relationships, and the limited response rate associated with a national phone sample. Further, the authors recognize the theoretical constructs in the risk communication literature can be better represented through more robustly constructed measures. However, in the interest of obtaining data rapidly to inform the ongoing public health efforts towards Zika, single item measures were used. Because of the limited number of cases in the United States, risk perception and knowledge may have been more limited than had there been a large Zika outbreak. In the event of a larger outbreak, health behaviors and attitudes may have appeared differently.

## Conclusions

In an emerging event with an absence of certainty, public receptivity to public health interventions may be associated with pre-existing social and political norms. Risk communicators should consider addressing these underlying constructs, with potentially targeted campaigns, in order to increase public acceptance of public health intervention strategies. Current risk models may be under-specified, particularly during periods of greatest scientific uncertainty, and they may over-estimate the value of knowledge, risk itself, or self-efficacy. This analytic approach explored an evolving risk in the context of traditional risk communication theory, which posits that a combination of knowledge, risk perception, and efficacy will impact behavior change. Knowledge and risk are represented by independent variables in the model and receptivity to each of the interventions described stands as a proxy for intended behavior change. However, in this case, the inconsistency in factors linked to intervention receptivity suggests a more nuanced understanding is necessary to understand the public’s appetite for behavior change in the face of the rising Zika epidemic.

## Supporting information

S1 File Zika Submission 6817.dta. (DTA)
